# NT-proBNP and exercise capacity in adult patients with congenital heart disease and a prosthetic valve: a multicentre PROSTAVA study

**DOI:** 10.1007/s12471-016-0896-5

**Published:** 2016-09-12

**Authors:** R. C. Schoonbeek, P. G. Pieper, Y. J. van Slooten, H. G. Freling, G. T. Sieswerda, A. P. J. van Dijk, M. R. M. Jongbloed, M. C. Post, B. J. Bouma, R. M. F. Berger, T. Ebels, J. P. van Melle

**Affiliations:** 1 Department of Cardiology, University Medical Center Groningen, University of Groningen, Groningen, The Netherlands; 2 Department of Thoracic Surgery, University Medical Center Groningen, University of Groningen, Groningen, The Netherlands; 3 Department of Radiology, University Medical Center Groningen, University of Groningen, Groningen, The Netherlands; 4 Interuniversity Cardiology Institute of the Netherlands, Utrecht, The Netherlands; 5 University Medical Center Utrecht, Utrecht, The Netherlands; 6 Radboud University Medical Center, Nijmegen, The Netherlands; 7 Leiden University Medical Center, Leiden, The Netherlands; 8 St. Antonius Hospital, Nieuwegein, The Netherlands; 9 Academic Medical Center, Amsterdam, The Netherlands

**Keywords:** Congenital heart disease, Pulmonary valve replacement, NT-proBNP

## Abstract

**Objectives:**

N-terminal B‑type natriuretic peptide (NT-proBNP) is an important biomarker for the detection of heart failure. Adults with congenital heart disease (ACHD) and a prosthetic heart valve are at risk for heart failure. This study aimed to determine the value of NT-proBNP in ACHD patients with a prosthetic valve and investigate its relationship with cardiac function and exercise capacity.

**Methods:**

In this multi-centre cross-sectional observational study, data regarding medical history, echocardiography, exercise testing (VO_2_peak) and laboratory blood evaluation (including NT-proBNP) were collected in ACHD patients with a single prosthetic valve (either homografts, heterografts or mechanical valves).

**Results:**

A total of 306 ACHD patients with pulmonary valve replacement (PVR, *n* = 139), aortic valve replacement (*n* = 141), mitral valve replacement (*n* = 21) or tricuspid valve replacement (*n* = 5) were investigated. The majority of patients (77 %) were in NYHA class I or II. Elevated NT-proBNP levels (cut-off ≥125 pg/ml) were found in 50 % of the patients, with the highest levels in patients with mitral valve replacements. In this study population, NT-proBNP levels were associated with gender (*p* = 0.029) and VO_2_max (*p* < 0.001). In PVR patients, NT-proBNP levels were associated with lower VO_2_peak, also after adjustment for age, gender and age at valve replacement in a multivariate model (*p* = 0.015).

**Conclusions:**

In patients with ACHD and a prosthetic valve, elevated NT-proBNP levels are frequently observed despite preserved NYHA class. In PVR patients, a higher NT-proBNP level was associated with a lower VO_2_peak. These results may be of importance in the ongoing discussion about the timing of valve replacement in patients with CHD.

## Introduction

N-terminal B‑type natriuretic peptide (NT-proBNP) is a well-established marker of heart failure (HF) and a predictor of impaired cardiovascular prognosis [1]. The value of NT-proBNP has been investigated in many patient populations, including patients with acute and chronic HF [2, 3] and patients with valvular heart disease [4]. Remarkably, data on NT-proBNP in adult patients with congenital heart disease (ACHD) are scarce. A cross-sectional study among patients with all types of ACHD showed that NT-proBNP levels were elevated in a substantial but variable percentage (26–88 %) [5], reflecting the heterogeneous nature of this population. Therefore, to define its clinical utility, further research is warranted among specific subgroups.

Within the diverse population of CHD patients, valve replacement surgery is often inevitable [6]. Recently, Diller et al. [7]. demonstrated that especially those patients with a history of valve replacement surgery are at risk for HF. This may be related to the fact that prosthetic valves are often implanted during childhood or adolescence, resulting in a probably higher prevalence of prosthesis patient mismatch (PPM) than in non-congenital patients [8]. In addition, also a watchful waiting strategy to allow children and adolescents to grow further, in order to implant a larger prosthetic valve, may result in valve-related HF and deteriorating functional class [9]. To what extent NT-proBNP levels are of value for the clinical evaluation of ACHD patients with a prosthetic valve is unclear. Therefore, the aim of this study was to investigate associations between NT-proBNP and characteristics of valve prosthesis (including PPM), ventricular function and exercise capacity in ACHD patients with a prosthetic valve.

## Methods

### Patient population

In this multi-centre cross-sectional observational study, named PROSTheses in Adult congenital heart VAlve disease (acronym: PROSTAVA), ACHD patients were selected from the Dutch CONCOR registry [10]. The PROSTAVA study was conducted to investigate the relationship between characteristics of valve prostheses (type, labelled size, location) and functional outcome in ACHD patients. Study design and rationale were previously described [11]. Briefly, patients with a prosthetic heart valve (homografts, heterografts and mechanical valves in aortic, mitral, pulmonary or tricuspid position) registered in the CONCOR database and followed in one of the six PROSTAVA centres were eligible. Patients with multiple prosthetic valves or transposition of the great arteries with a systemic right ventricle, either congenitally corrected or after Mustard/Senning correction, were excluded. The study was conducted in accordance with the Declaration of Helsinki and was approved by the institutional ethics committees.

### Collected data

Collected data concerned demography and medical history, including previous cardiac surgical interventions, and were obtained by reviewing patient records. Prospective data were collected during routine clinical care: echocardiography, exercise testing and laboratory blood tests (including NT-proBNP and renal function).

### NT-proBNP

Measurement of NT-proBNP was performed using the Elecsys proBNP ELISA (Roche Diagnostics, Mannheim, Germany). NT-proBNP was considered high when levels were ≥125 pg/ml, based on the European Society of Cardiology guidelines for non-acute presentation of heart failure [12].

### Echocardiography

Routine two-dimensional, M‑mode and Doppler echocardiography were performed by experienced, board-certified sonographers and supervised by cardiologists of the participating hospital. Both the images and reports were sent to the University Medical Center Groningen, where the echocardiograms were blinded. Measurements related to the prosthetic valve were independently re-assessed and subsequently checked by senior investigators.

Other measurements concerning ventricular size and function and native valve morphology and function were collected from the original echo reports. Left ventricular ejection fraction (LVEF) was measured using Simpson’s rule or, when image quality was suboptimal, using visual estimation. Right ventricular function was assessed with tricuspid annular plane systolic excursion (TAPSE). Prosthetic valvar effective orifice area (EOA in cm^2^) was calculated using the continuity equation [13]. EOA was indexed for body surface area (iEOA). For aortic valve replacement (AVR) patients, PPM was classified as moderate PPM (iEOA 0.85–0.65 cm^2^/m^2^) and as severe PPM (iEOA <0.65 cm^2^/m^2^) [8]. The native pulmonary EOA in adults is about 30 % larger than the aortic EOA [14]. We therefore assumed cut-off values for PPM in pulmonary valve replacement (PVR) patients accordingly (moderate PPM was defined as iEOA ≤1.10 cm^2^/m^2^ and severe PPM as iEOA ≤0.85 cm^2^/m^2^).

### Exercise capacity testing

Exercise capacity was measured using a bicycle or treadmill ergometer, assessing either peak oxygen uptake (VO_2_peak, ml/min/kg) or maximum workload (Wpeak, Watts). The predicted peak oxygen uptake (VO_2_pred) and workload (Wpred) was calculated as previously described [15, 16].

Exercise capacity testing was performed uniformly and reproducible among the different centres and started with a short warm-up (without load), followed by a stepwise increase of workload consistent with a protocol based on sex, age, height and weight. Exercise duration was aimed for at least 6–8 minutes. The percentage of predicted exercise capacity (PPEC) was calculated by dividing the achieved exercise level (VO_2_peak or Wpeak) by the predicted exercise level (VO_2_pred or Wpred). A PPEC ≤75 % was considered a decreased exercise capacity [17].

### Statistics

Data were analysed using SPSS® Statistics version 22.0. Descriptive statistics were calculated as mean values and standard deviations for normally distributed continuous variables, medians and quartiles for continuous variables with non-normal distribution and absolute numbers and percentages for dichotomous variables. Comparisons of continuous variables between groups were made by unpaired Student’s t‑tests or the Mann-Whitney U test, depending on their distribution, and dichotomous variables were compared using the χ^2^ or Fisher’s exact test. Univariable and multivariable regression analysis (dependent variable: NT-proBNP) was used to study the association between NT-proBNP and prosthetic valve characteristics or functional capacity. All independent covariates with *p* < 0.10 in univariable regression analyses were included in multivariable regression analysis. A two-sided *p*-value <0.05 was considered statistically significant. Due to the limited number of patients with mitral valve replacement (MVR) and tricuspid valve replacement (TVR), regression analyses were confined to AVR and PVR patients.

## Results

**Fig. 1:**
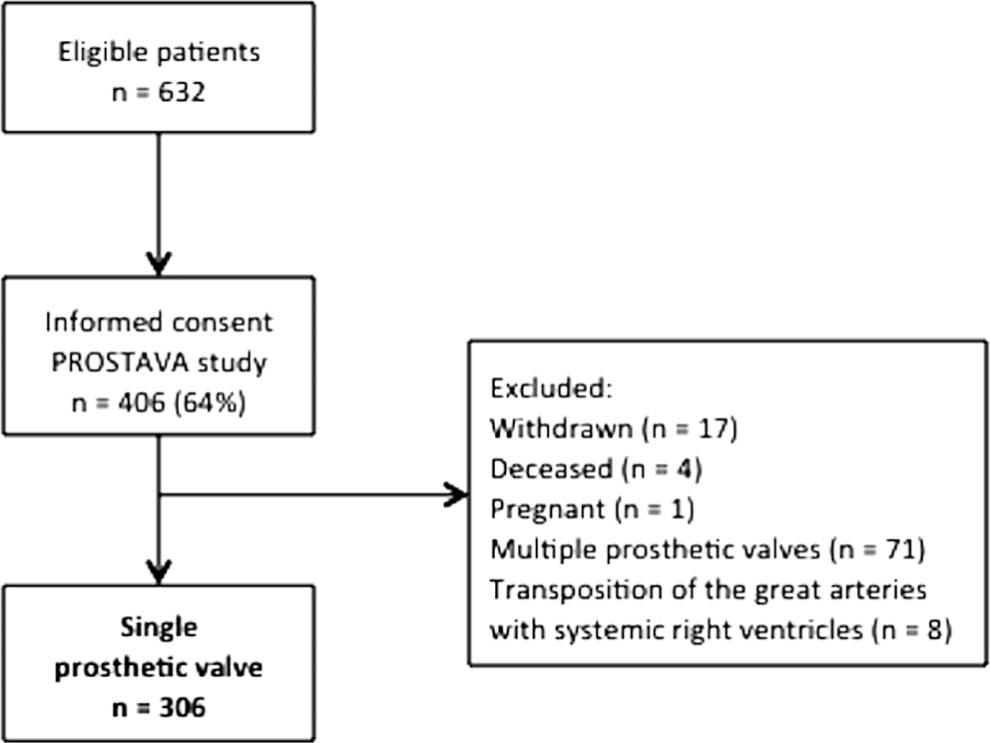
Flowchart of patient inclusion

A flowchart for patient selection with exclusion criteria is shown in Fig. 1. The study population comprised 141 AVR patients, 139 PVR patients, 21 MVR patients and 5 TVR patients. Baseline characteristics are presented in Table 1. The majority were male (57 %). The main diagnosis in PVR patients was Tetralogy of Fallot (ToF, 66 %) and in AVR patients bicuspid aortic valve (49 %).

**Table 1:** Baseline characteristics

	Total (*n* = 306)	AVR (*n* = 141)	PVR (*n* = 139)	MVR (*n* = 21)	TVR (*n* = 5)
Male (%)	174 (57)	95 (67)	70 (50)	8 (38)	1 (20)
*Diagnosis (%)*					
Tetralogy of Fallot	–	–	66	–	–
Pulmonary atresia	–	–	12	–	–
Pulmonary valve stenosis	–	–	12	–	–
Bicuspid aortic valve	–	49	–	–	–
(Sub)valvular aortic stenosis	–	25	–	–	–
Marfan syndrome	–	19	–	–	–
AVSD	–	–	–	48	–
Mitral stenosis	–	–	–	19	–
Ebstein malformation	–	–	–	–	100
Other	–	7^1^	10^2^	33^3^	–
*Indication for valve replacement (%)*					
Regurgitation	–	29	70	38	20
Stenosis	–	21	13	14	–
Regurgitation and stenosis	–	20	11	14	–
Other indications^4^	–	30	6	34	80
Age at first VR (y, SD)	27 ± 13	30 ± 11	24 ± 13	25 ± 17	19 ± 11
Age at last VR (y, SD)	30 ± 12	33 ± 11	26 ± 11	31 ± 13	29 ± 13
Time since last VR (y, SD)	9 ± 7	11 ± 8	8 ± 6	10 ± 6	3 ± 4
*Number of valve replacements (%)*					
1	232 (76)	105 (74)	114 (82)	12 (57)	1 (20)
2	59 (19)	29 (21)	19 (14)	8 (38)	3 (60)
3	14 (5)	6 (4)	6 (4)	1 (5)	1 (20)
4	1 (0)	1 (1)	0 (0)	0 (0)	0 (0)
*Valve type (%)*					
*Biological*	133 (43)	15 (11)	114 (82)	1 (5)	3 (60)
Bio conduit	5 (4)	0 (0)	5 (4)	0 (0)	0 (0)
Bio stented	111 (83)	7 (47)	101 (73)	1 (100)	2 (67)
Bio stentless	11 (8)	8 (53)	3 (2)	0 (0)	0 (0)
Transcatheter	7 (5)	0 (0)	6 (4)	0 (0)	1 (33)
*Mechanical*	170 (56)	126 (89)	22 (16)	20 (95)	2 (40)
Bi-leaflet	154 (90)	113 (90)	22 (100)	17 (85)	2 (100)
Mono-leaflet	16 (9)	13 (10)	0 (0)	3 (15)	0 (0)
Valve size	25 ± 3	23 ± 3	25 ± 2	29 ± 3	31 ± 4
Age (y, SD)	39 ± 11	44 ± 11	35 ± 10	41 ± 13	32 ± 13
Body surface area (SD)	1.95 ± 0.2	1.99 ± 0.2	1.92 ± 0.2	1.87 ± 0.2	1.87 ± 0.3
BMI (SD)	24.7 ± 4	25.1 ± 4	24.2 ± 4	24.7 ± 4	27.5 ± 4
*NYHA (%)*					
I	148 (48)	82 (58)	65 (47)	1 (5)	0 (0)
II	86 (28)	41 (29)	42 (30)	3 (14)	0 (0)
III	42 (14)	10 (7)	12 (9)	15 (71)	5 (100)
IV	4 (1)	0 (0)	2 (1)	2 (10)	0 (0)
Unknown	26 (9)	8 (6)	18 (13)	0 (0)	0 (0)

^1^ Truncus arteriosus (2.9); atrial septal defect + D.B. (0.7); ventricular septal defect (VSD) (0.7); double outlet right ventricle (DORV) Fallot (0.7); DORV transposition of the great arteries (TGA) (0.7); TGA + pulmonary stenosis + VSD (2.2); empty cells (2.2).

^2^VSD + coarctation (3.6); TGA (1.4); PA + VSD (0.7).

^3^Anomalous left coronary artery arising from the pulmonary artery (ALCAPA) (4.8); ASD (21.3); DORV + coarctation (4.8); Marfan (4.8); bicuspid aortic valve + coarctation (4.8).

^4^ Other indications include valve atresia, aorta dissection, aortic dilatation, endocarditis and valve complications. Values are mean/median ± standard deviation (SD); *AVR* aortic valve replacement, *PVR* pulmonary valve replacement, *MVR* mitral valve replacement, *TVR* tricuspid valve replacement, *VR* valve replacement, *AVSD* atrioventricular septal defect, *VSD* ventricular septal defect, *TGA* transposition of the great arteries, *PA* patent arteriosus, *ToF* Tetralogy of Fallot, *ASD* atrial septal defect, *DORV (Fallot)* double outlet right ventricle (ToF), *ALCAPA* anomalous left coronary artery arising from the pulmonary artery, *NYHA* New York Heart Association, *y* years.

Mean age at surgery for first and latest valve replacement was 27 ± 13 (range 0–51) and 30 ± 12 years (range 3–67), respectively. The valve type differed between AVR and PVR patients: 89 % of AVR were mechanical valves while in PVR 82 % of the patients had biological valves implanted (*p* < 0.001). The majority of patients were in NYHA class I–II at inclusion (76 %). NYHA class was significantly higher in MVR patients compared with PVR and AVR patients (*p* < 0.001). Age at first and last valve replacement was significantly higher in AVR patients compared with PVR patients (*p* < 0.001).

### NT-proBNP

**Fig. 2:**
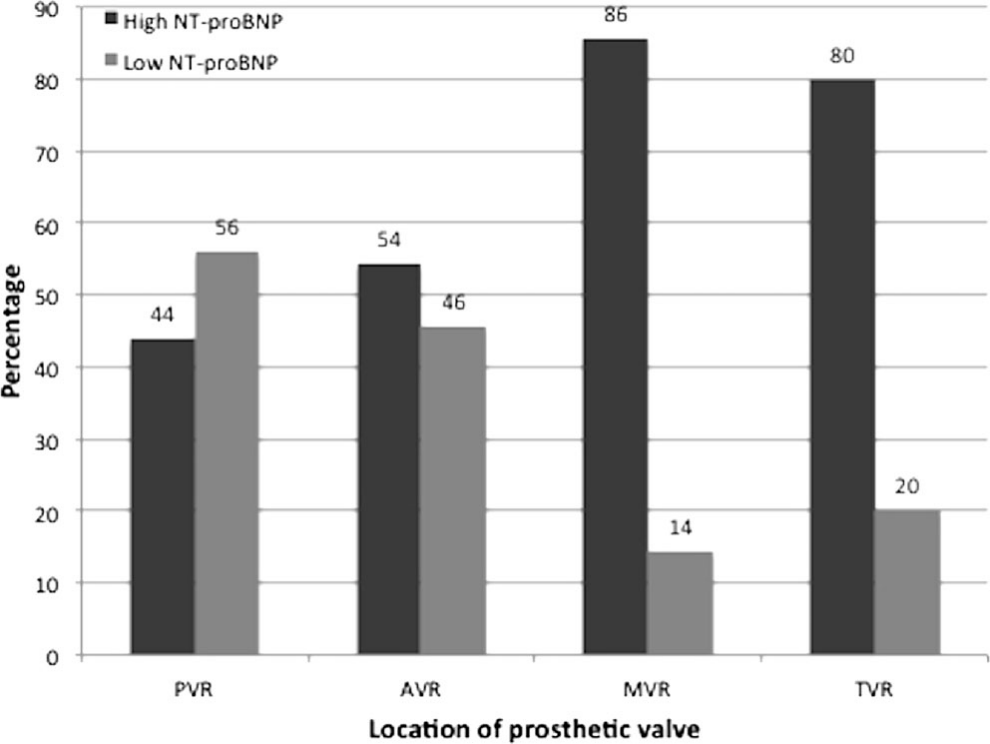
NT-proBNP: percentages elevated NT-proBNP (≥125 pg/ml) and low NT-proBNP (<125 pg/ml) in different valve locations; *NT-proBNP* N‑terminal pro-brain natriuretic peptide, pg/ml

The median NT-proBNP level in the total study population was 140 (range 13–3132) pg/ml (Table 2). Patients with higher NT-proBNP levels were more likely to be women (median level 183 and 104 pg/ml in women and men, respectively, *p* < 0.001) and older (*p* < 0.001). For the whole study sample, higher age at latest valve replacement was significantly associated with higher NT-proBNP levels (*p* = 0.02). Median NT-proBNP level in AVR patients was 146 pg/ml, compared with 108 pg/ml in PVR patients (*p* = ns). The highest NT-proBNP levels were seen in MVR patients (median 316 pg/ml; *p* < 0.001 and *p* = 0.002 compared with PVR and AVR, respectively). High NT-proBNP levels (≥125 pg/ml) were found in 44 % of the PVR patients, 54 % of the AVR patients, and 86 and 80 % of the MVR and TVR patients, respectively (Fig. 2).

**Table 2:** Echocardiography, exercise capacity and NT-proBNP

Method	Variable	Total	AVR	PVR	MVR	TVR
	*n* (%)	306 (100)	141 (46)	139 (45)	21 (7)	5 (2)
Echocardiography	LVEF (%)	55 ± 8	57 ± 9	55 ± 6	50 ± 11	–
	LVEF ≥50 % (*n*, %)	108 (77)	35 (78)	70 (78)	3 (60)	–
	LVEDD (mm)	50 ± 8	50 ± 7	49 ± 8	53 ± 10	48 ± 7
	LVESD (mm)	34 ± 8	33 ± 7	34 ± 7	41 ± 9	28 ± 7
	LV mass (g/m^2^ BSA)	97 ± 33	98 ± 29	60 ± 54	112 ± 40	80 ± 28
	TAPSE (mm)	17 ± 4	17 ± 4	17 ± 4	18 ± 3	15 ± 3
	TAPSE ≥17 mm (*n*, %)	150 (56)	59 (54)	75 (57)	14 (70)	2 (40)
	EOA (cm^2^/m^2^)	2.1 ± 1	1.9 ± 1	2.4 ± 2	2.4 ± 1	4.9
Exercise testing	VO_2_peak (ml/min/kg)	27 ± 8	29 ± 8	28 ± 7	22 ± 7	19 ± 6
	PPEC (%)	81 ± 23	84 ± 24	76 ± 19	72 ± 25	71 ± 34
	PPEC ≤75 % (*n*, %)	94 (41)	32 (31)	48 (47)	12 (67)	2 (50)
Laboratory blood	NT-proBNP	140 ± 329	146 ± 374	108 ± 253	316 ± 372	247 ± 300
	% High NT-proBNP	52	54	44	86	80

Values are mean or median ±SD or *n*. *LVEF* left ventricular ejection fraction, *LVEDD* left ventricular end-diastolic diameter, *LVESD* left ventricular end-systolic diameter, *RVEF* right ventricular ejection fraction, *TAPSE* tricuspid annular plane systolic excursion, *EOA* effective orifice area, *PPEC* percentage of predicted exercise capacity, *NT-proBNP* N-terminal pro-brain natriuretic peptide, pg/ml (cut-off for high NT-proBNP ≥125 pg/ml)

### Echocardiography and exercise testing

Echocardiographic characteristics of the entire study population and for each separate prosthetic valve location are displayed in Table 2. Normal LVEF was found in 78 % of the AVR patients and 78 % of the PVR patients. Left ventricular (LV) mass was higher in AVR in comparison with PVR patients (*p* = 0.015).

A normal right ventricular function (TAPSE ≥17 mm) was found in 56.4 % (*n* = 150) of all patients, including 57.3 % of PVR patients (*n* = 75) and 53.6 % of AVR patients (*n* = 59). The effective orifice area (EOA) of the pulmonary valves was significantly larger than aortic prosthetic valves (Table 2, *p* = 0.003). PPM was found in 50 % of the PVR patients and 48 % of the AVR patients.

Exercise capacity was assessed in 228 patients (75 %), with 194 VO_2_max tests (85 %) and 34 ergometric tests (15 %). In all patients, the median PPEC was 81 % (IQR 65–98 %) and 41 % had impaired exercise capacity (PPEC ≤75 %, Table 2). Decreased PPEC was observed in 31 % of the AVR patients, compared with 47 % of the PVR patients (*p* = 0.017). VO_2_peak was significantly lower in MVR compared with AVR (*p* = 0.001) and PVR patients (*p* < 0.001).

### Associations with high NT-proBNP levels (≥125 pg/ml)

For the entire study population, gender (*p* < 0.001), age (*p* < 0.001), age at last valve replacement (*p* = 0.020), renal function (*p* = 0.022) and VO_2_max (*p* < 0.001) were associated with NT-proBNP (*p* < 0.05). Gender (*p* = 0.029) and VO_2_max (*p* < 0.001) were associated with NT-proBNP in multivariate analysis as well (Table 3).

**Table 3:** Univariable and multivariable parameters associated with NT-proBNP for all patients (cut-off 125 pg/ml)

	Univariate		Multivariate	
	All		All	
Variable	95 % CI (B)	*p*	95 % CI (B)	*p*
BSA (m^2^)	−0.50 to 0.07	0.141	–	**–**
BMI (kg/m^2^)	−0.01 to 0.02	0.545	–	–
Gender	−0.41 to −0.15	**>0.001**	−0.34 to −0.02	**0.029**
Age	0.01 to 0.02	**>0.001**	−0.01 to 0.02	0.310
NYHA class	−0.01 to 0.16	**0.077**	−0.08 to 0.11	0.804
Age at first VR (y)	0.00 to 0.01	0.164	–	–
Age at last VR (y)	0.00 to 0.01	**0.020**	−0.02 to 0.01	0.295^*^
Time since last VR (y)	0.00 to 0.02	**0.093**	–	–
Valve size (mm)	0.01 to 0.03	0.434	–	–
Valve type (bio/mech)	0.00 to 0.27	**0.055**	−0.07 to 0.25	0.257
iEOA (cm^2^/m^2^)	−0.18 to 0.12	0.689	–	–
PPM (iEOA ≤0.85 cm^2^/m^2^)	−0.21 to 0.11	0.520	–	–
Indexed LV mass (g/m^2^)	0.00 to 0.00	0.313	–	–
LVEF (%)	−0.01 to 0.01	0.939	–	–
LVEDD (mm)	−0.01 to 0.01	0.402	–	–
LVESD (mm)	0.00 to 0.02	0.184	–	–
RVEF (%)	−0.01 to 0.02	0.371	–	–
TAPSE (mm)	−0.02 to 0.02	0.808	–	–
Renal function (ml/min)	−0.01 to 0.00	**0.022**	−0.01 to 0.00	0.107
VO_2_max (ml/min/kg)	−0.03 to −0.02	**<0.001**	−0.03 to −0.01	**<0.001**
PPEC (%)	−0.01 to 0.00	0.113	–	–

*CI* confidence interval, *BSA* body surface area, *BMI* body mass index, *VR* valve replacement, *iEOA* indexed effective orifice area, *PPM* patient prosthesis mismatch, *LVEF* left ventricular ejection fraction, *LVEDD* left ventricular end-diastolic diameter, *LVESD* left ventricular end-systolic diameter, *RVEF* right ventricular ejection fraction, *TAPSE* tricuspid annular plane systolic excursion, *PPEC* percentage of predicted exercise capacity, *np* not performed. Bold text: univariable parameters with a *p*-value <0.1 are used in multivariate regression analysis. ^*^Collinearity of Statistics is reported between the variables ‘age at last VR’ and ‘time since last VR’

**Fig. 3:**
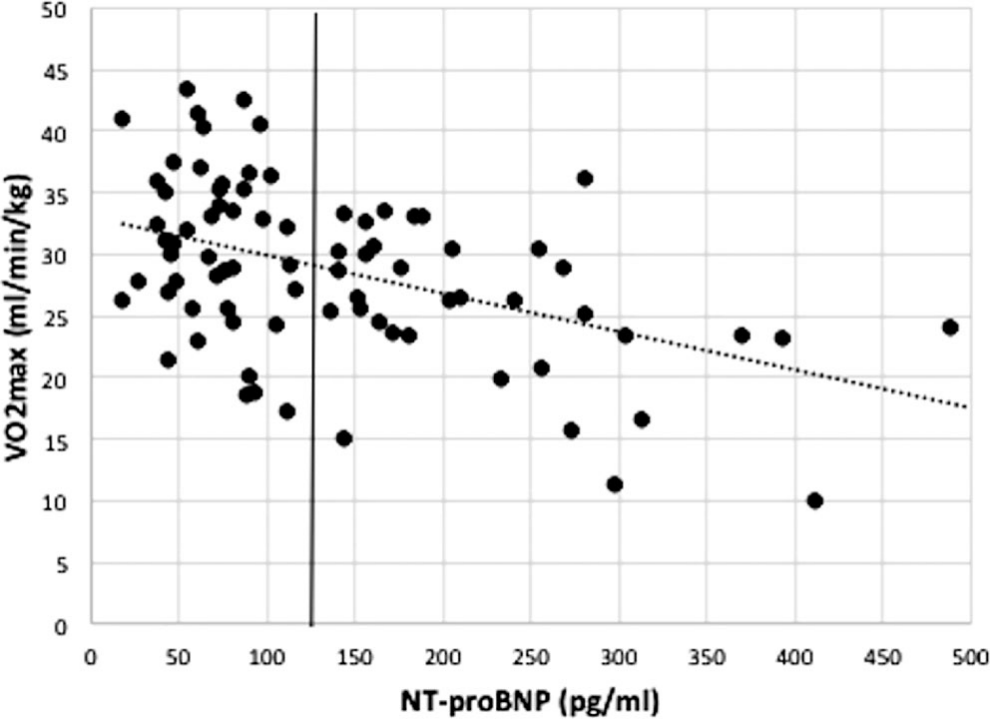
Association between NT-proBNP (cut-off 125 pg/ml) and VO_2_peak in PVR patients; *NT-proBNP* N-terminal pro-brain natriuretic peptide, pg/ml (cut-off for high NT-proBNP ≥125 pg/ml), *PVR* pulmonary valve replacement. Dotted line indicates cut-off for elevated NT-proBNP levels (125 pg/ml). Pearson’s r = −0.555, *p* < 0.001, y = 33 − 0.03*x

In the PVR group, univariable parameters associated with NT-proBNP (*p* < 0.1) included age at valve replacement surgery (*p* = 0.091) and VO_2_peak (*p* < 0.001) (Table 4; Fig. 3). In a multivariate model, a higher NT-proBNP level remained significantly associated with a lower VO_2_peak, even after adjustment for age, gender and age at valve replacement surgery (*p* = 0.015), as presented in Table 4. PPEC was negatively associated with NT-proBNP as well (*p* = 0.025 in PVR) when corrected for the same variables.

**Table 4:** Univariable and multivariable parameters associated with NT-proBNP for PVR and AVR patients (cut-off 125 pg/ml)

	Univariate				Multivariate			
	PVR		AVR		PVR		AVR	
Variable	95 % CI (B)	*P*	95 % CI (B)	*P*	95 % CI (B)	*P*	95 % CI (B)	*P*
BSA (m^2^)	−0.59 to 0.27	0.460	−0.76 to 0.10	0.128	–	–	–	–
BMI (kg/m^2^)	−0.02 to 0.02	0.990	−0.03 to 0.03	0.877	–	–	–	–
Gender	−0.47 to −0.08	**0.007**	−0.52 to −0.14	**0.001**	−0.43 to −0.01	**0.045**	−0.57 to −0.02	**0.034**
Age	0.01 to 0.03	**0.004**	0.00 to 0.02	**0.047**	−0.00 to 0.05	0.098	−0.01 to 0.01	0.951
NYHA class	−0.10 to 0.18	0.582	−0.13 to 0.18	0.769	–	–	–	–
Age at first VR (y)	−0.00 to 0.01	0.119	−0.01 to 0.01	0.710	–	–	–	–
Age at last VR (y)	−0.00 to 0.02	**0.091**	−0.00 to 0.01	0.247	−0.03 to 0.01	0.251	–	–
Time since last VR (y)	−0.01 to 0.03	0.174	−0.01 to 0.02	0.258	–	–	–	–
Valve size (mm)	−0.04 to 0.05	0.811	−0.06 to 0.01	0.172	–	–	–	–
Valve type (bio/mech)	−0.15 to 0.36	0.397	−0.32 to 0.41	0.802	–	–	–	–
iEOA (cm^2^/m^2^)	−0.22 to 0.19	0.882	−0.37 to 0.19	0.523	–	–	–	–
PPM (iEOA ≤0.85 cm^2^/m^2^)	−0.10 to 0.44	0.204	−0.23 to 0.18	0.816	–	–	–	–
Indexed LV mass (g/m^2^)	Np	Np	−0.00 to 0.01	0.217	–	–	–	–
LVEF (%)	−0.01 to 0.02	0.503	−0.03 to 0.01	0.440	–	–	–	–
LVEDD (mm)	−0.03 to 0.01	0.217	−0.01 to 0.02	0.250	–	–	–	–
LVESD (mm)	−0.02 to 0.02	0.735	−0.01 to 0.02	0.421	–	–	–	–
RVEF (%)	−0.01 to 0.02	0.666	−0.01 to 0.02	0.641	–	–	–	–
TAPSE (mm)	−0.03 to 0.02	0.859	−0.03 to 0.03	0.912	–	–	–	–
Renal function (ml/min)	−0.01 to 0.00	0.248	−0.01 to −0.00	**0.009**	–	–	−0.01 to 0.00	0.087
VO_2_max (ml/min/kg)	−0.04 to −0.02	**<0.001**	−0.03 to −0.00	**0.042**	−0.04 to −0.00	**0.015**	−0.02 to 0.01	0.698
PPEC (%)	−0.01 to 0.00	0.245	−0.01 to 0.00	0.922	–	–	–	–

*AVR* aortic valve replacement,* PVR* pulmonary valve replacement,* CI* confidence interval, *BSA* body surface area, *BMI* body mass index, *VR* valve replacement, *iEOA* indexed effective orifice area, *PPM* patient prosthesis mismatch, *LVEF* left ventricular ejection fraction, *LVEDD* left ventricular end-diastolic diameter, *LVESD* left ventricular end-systolic diameter, *RVEF* right ventricular ejection fraction, *TAPSE* tricuspid annular plane systolic excursion, *PPEC* percentage of predicted exercise capacity, *np* not performed. Bold text: univariable parameters with a *p*-value <0.1 are used in multivariate regression analysis

In AVR patients, VO_2_peak was negatively associated with NT-proBNP as well, although in a multivariable model, including age, gender and renal function, only gender remained significantly associated with NT-proBNP (*p* = 0.034, Table 4).

## Discussion

This multi-centre cross-sectional study focused on the value of NT-proBNP in ACHD and a prosthetic valve. Using the European Society of Cardiology cut-off for chronic HF of 125 pg/ml, elevated NT-proBNP levels were found in more than 50 % of the patients. In PVR patients, high NT-proBNP levels were associated with decreased exercise capacity.

### NT-proBNP in CHD

The high percentage of elevated NT-proBNP levels in PVR patients and AVR patients (44 and 54 %, respectively) is remarkable. In a smaller subgroup of patients with an atrioventricular prosthetic valve, this percentage was even higher (i. e. 86 % for MVR and 80 % for TVR patients). The high percentages of elevated NT-proBNP levels in our ACHD population are comparable to, for example, populations with stable chronic kidney disease (127 vs. 140 pg/ml in this population) [18], but not as high as recently reported in a population with HF with preserved ejection fraction (median 1772 pg/ml) [19]. For ACHD patients, Eindhoven et al. found comparable percentages of elevated NT-proBNP levels in both a heterogeneous group with various ACHD patients [5] and in adult patients with corrected ToF (53 and 55 %, respectively) [20]. However, in their study population with various CHD lesions, patients with systemic right ventricular and Fontan circulation mainly contributed to the elevated NT-proBNP levels. In our study, these patients were excluded.

The higher NT-proBNP levels found in AVR patients compared with PVR patients (146 vs. 108 pg/ml, respectively) could possibly be explained by the higher LV mass found in AVR patients (*p* = 0.015). The study by Mishra et al. [18]. in patients with chronic kidney disease describes the association between high NT-proBNP levels and higher LV mass. However, this association between high NT-proBNP levels and LV mass in AVR patients did not hold true in the regression analysis.

Whether the high percentages of elevated NT-proBNP in our study cohort are related to surgical technique, prosthetic valve material, (subclinical) heart failure or the timing of valve surgery could not be answered by this study. Serial measurements could shed light on this interesting question.

### Associations with high NT-proBNP levels (≥125 pg/ml)

In line with other studies, NT-proBNP levels were higher in women and associated with ageing [21]. This finding highlights the need for sex- and age-specific reference values of NT-proBNP to be useful for diagnostic purposes.

Although NT-proBNP levels tended to be related to the age at last valve replacement, indicating that higher age at last valve replacement may possibly result in higher NT-proBNP levels at adult age, this association did not hold true after adjustment for sex and age. At least in our population, this may indicate that high pro-BNP levels are not a reflection of a late valve replacement. Given the relationship between ventricular dysfunction and HF, it may be anticipated that ventricular function is associated with NT-proBNP levels. Yet, we did not find such a relationship. However, diastolic characteristics (not taken into account in our study) are also of importance in the release of NT-proBNP, as recently described for a CHD population [22]. Although we previously found an association between PPM and exercise capacity in AVR patients [23], this did not translate into higher NT-proBNP levels in this subgroup. At least for AVR patients, a possible explanation could be the rather preserved left ventricular systolic function (mean LVEF 57 %).

### NT-proBNP and exercise capacity in PVR patients

We found an association between NT-proBNP and exercise capacity in PVR patients: a higher NT-proBNP level was associated with a lower VO_2_peak. This result is in line with the findings in patients with various CHD lesions (with or without prosthetic valve) of Eindhoven et al. [5]. Of note, Eindhoven et al. could not establish this association in their population of adult ToF patients [20]; however in their study only 55 % of the ToF patients had a prosthetic valve, whereas our findings were most profound in PVR patients, of which 66 % were diagnosed with ToF. Our results are in line with those of Norozi et al. who found a correlation between exercise capacity and NT-proBNP in ToF patients, also showing comparable NT-proBNP levels as found in our study [24]. Our findings strengthen the use of NT-proBNP as possible marker of functional capacity in PVR patients. Although right ventricular function is an important determinant of exercise performance [25], we could not confirm significant associations between NT-proBNP and TAPSE. However, one has to keep in mind that TAPSE reflects only longitudinal right ventricular contraction, whereas radial contraction may be preserved or even enhanced postoperatively. This finding is reinforced by the fact that in AVR patients TAPSE was decreased in a similar proportion (46 % vs. 43 % in PVR patients, *p* = ns).

## Limitations

The multiple types and locations of the prosthetic valves in combination with the underlying cardiac malformation resulted in a heterogeneous population. We chose to analyse our data separately for each valve location. Therefore, the study results that were found in PVR patients could not be extrapolated to patients with another located prosthetic valve (or vice versa). Finally, due to the limited number of patients, MVR and TVR patients were not included in the regression analysis.

## Conclusion

NT-proBNP levels are elevated in more than 50 % of the ACHD population with a prosthetic valve. Higher NT-proBNP levels are strongly associated with poor exercise capacity in PVR patients. Further, prospective studies of NT-proBNP values in CHD patients are necessary in order to assess its predictive value for prognosis in these patients.
